# Health Care Use > 2 Years After Cancer Diagnosis: Results From a Survey Among 5,710 Patients With Various Cancer Types

**DOI:** 10.1002/pon.70407

**Published:** 2026-02-14

**Authors:** Floortje Mols, Noortje van Willegen, Dagna Lek, Vivian Engelen

**Affiliations:** ^1^ Department of Medical and Clinical Psychology CoRPS ‐ Center of Research on Psychological Disorders and Somatic Diseases Tilburg University Tilburg the Netherlands; ^2^ Department of Research & Development Netherlands Comprehensive Cancer Organization (IKNL) Utrecht the Netherlands; ^3^ Dutch Federation of Cancer Patients Organizations Nederlandse Federatie van Kankerpatiëntenorganisaties (NFK) Utrecht the Netherlands

**Keywords:** cancer, health care use, oncology, survivorship

## Abstract

**Purpose:**

This study aimed to examine health care use in a cross‐sectional sample of Dutch cancer survivors > 2 years post‐diagnosis.

**Methods:**

The Dutch Federation of Cancer Patient Organizations (NFK), together with patient representatives and researchers, developed a cross‐sectional online survey on life after cancer, which was distributed via email, websites, and social media.

**Results:**

The study included 5710 respondents (> 2 years post‐diagnosis). Among those who reported long‐term cancer/treatment‐related consequences (approximately 89% of participants), one‐third (33%) had received professional care or support for these issues in the past 3 months. Those reporting more cancer‐ or treatment related consequences, those diagnosed 2–5 years ago, those (probably) not (getting) better, and those currently under treatment were more likely to receive professional care or support. Care was primarily provided by medical specialists (47%), physical therapists (37%), and/or general practitioners (32%). 15% expressed a desire for care or support that they did not receive, indicating reasons such as a long time since diagnosis or affordability. Overall, 68% knew where to turn for help; among those with consequences, 19% received peer/volunteer support and 10% wanted it but did not receive it.

**Conclusion:**

A significant proportion of long‐term cancer survivors in our sample reported unmet support needs (15% for professional care, 10% for peer support). Efforts should focus on improving access to affordable professional care, expanding peer support networks, providing personalized long‐term follow‐up care, and reducing stigma around seeking help, particularly within the context of the Dutch healthcare system.

**Abbreviations**
DJE: Donate Your Experience panel
IKNL: Netherlands Comprehensive Cancer Organization
NFK: Dutch Federation of Cancer Patient Organizations
WMO: Medical Research Involving Human Subjects Act

## 1 Background

Cancer survivorship is increasingly recognized as a distinct phase of the cancer care continuum, where individuals experience not only the physical challenges of cancer and cancer treatments but also long‐term psychological, social, and functional consequences [1]. With advancements in early detection, treatment, and improved survival rates, the focus of oncology care is shifting toward managing the late effects of cancer and its treatments. These sequelae can have profound impacts on survivors' quality of life, and therefore, adequate post‐treatment care and support are essential to help individuals manage these challenges [2]. Despite the importance of addressing these needs, access to appropriate care and the knowledge of where to seek support remain significant barriers for many cancer survivors [3].

Previous studies have shown that cancer‐related sequelae, such as fatigue, pain, mobility issues, and cognitive impairments, continue to affect a substantial proportion of cancer survivors years after treatment [4]. These long‐term effects can be under‐recognized, and patients may not always be aware of the professional help available to them. Furthermore, various socio‐demographic factors such as age, education, gender, and time since diagnosis can influence both the experience of cancer‐related consequences and the likelihood of searching for and receiving care or support [5, 6]. Addressing the needs of cancer survivors requires a comprehensive approach, not only through healthcare professionals but also by leveraging peer support networks, volunteer programs, and patient education [7].

In the Netherlands, various organizations, such as cancer patient organizations united in the Dutch Federation of Cancer Patient Organizations (NFK), kanker.nl, the Dutch Cancer Society, IPSO, and Netherlands Comprehensive Cancer Organisation (IKNL), work together to support those living with or after cancer, offering information on available care and peer connections. However, the extent to which cancer survivors are aware of or able to access these services remains unclear [8, 9, 10]. Although professional care and support is available in the Netherlands on a large scale [11], ensuring equitable long‐term support, and the organization of survivorship care remains a challenge, as highlighted in recent reviews [12, 13]. A crucial element in improving aftercare is understanding the specific needs and barriers survivors face when seeking professional care or support for the consequences of cancer. The present study aims [1] to explore which professional care cancer survivors utilize to address their cancer or treatment related consequences and [2] further needs and barriers in finding this care. Both goals can eventually enhance patient education efforts and can give insight into ways to improve the overall quality of post‐cancer care delivery. By drawing attention to the need for, and availability and accessibility of professional care and support that help mitigate the impact of cancer's aftermath, the study hopes to contribute to the development of more accessible, and patient‐centered care for cancer survivors in the Netherlands and beyond.

## 2 Methods

### 2.1 Study Design and Participants

For this study, a cross‐sectional online Dutch questionnaire was created (as part of NFK's Doneer Je Ervaring initiative) to explore the needs and experiences of cancer patients who had been diagnosed with cancer ≥ 2 years prior. Data collection was conducted via the Survalyzer platform. The survey was open for two weeks in the fall of 2023, promoted through the NFK website (https://doneerjeervaring.nl/) and various social media channels, including both sponsored and organic posts on Facebook and Instagram, as well as organic posts on LinkedIn and Twitter/X. Additionally, many cancer patient organizations affiliated with NFK emailed their members and donors with an invitation to participate, and also utilized their social media networks for recruitment. Members of the Donate Your Experience (DJE) panel were also sent email invitations. Further recruitment was carried out through partner organizations, including the Dutch Cancer Society, Kanker.nl, IPSO Centers for living with or after cancer, and several hospitals via their own communication channels.

In compliance with the General Data Protection Regulation (EU), all participants were informed about privacy policies and all provided informed consent. Since this study did not include an intervention, the Medical Research Involving Human Subjects Act (WMO) was not applicable, and ethical approval was not required.

### 2.2 Questionnaire

The questionnaire was co‐designed by a multi‐stakeholder team, including patient representatives from five cancer patient organizations, a patient advocate, a researcher from NFK, a consultant from IKNL, a researcher from the PROFILES registry, and a communications advisor from the Dutch Cancer Society. The content underwent iterative review for face and content validity within the team, resulting in a refined instrument. No separate pilot test was performed; instead, the iterative development with experts and patient representatives was used to ensure clarity and relevance of the questions.

In addition to essential socio‐demographic questions, participants were asked to self‐report their cancer type by selecting it from a predefined list, and to indicate in which year they were diagnosed. Those who reported being diagnosed < 2 years ago were excluded from further participation. We focused on those that confirmed the following question: “Do you suffer from physical or mental symptoms or problems due to (the treatment of) cancer?” (e.g., fatigue, neuropathy, anxiety; multiple answers possible). Key questions for this study included, among others; “Are you currently receiving professional care or support for the consequences of (the treatment of) cancer?”. If answered positively: “From whom do you receive this professional care?”. If answered negatively; “What is the reason?”. And for everybody: “Is it clear which professional care provider you can turn to if (the treatment of) cancer causes (new) consequences?”.

The anonymous questionnaire included multiple‐choice, numeric, and open‐ended items, with an average completion time of ∼15 min. Except for open‐ended questions, all questions were mandatory. Open‐ended responses were included to allow respondents to elaborate on their answers; these responses were not subjected to formal qualitative analysis in the current study. Respondents could pause and continue any time.

### 2.3 Statistical Analysis

Statistical analyses were conducted using IBM SPSS Statistics version 28. Categorical variables are presented as frequencies and percentages. Subgroup differences by gender, age at diagnosis, education level, time since diagnosis, disease phase, treatment status, and cancer type were tested using Pearson's chi‐square. Given the non‐probability sampling strategy and the primarily descriptive aim of this study, analyses were intentionally limited to bivariate comparisons. Applying multivariable regression models in this highly selected sample could suggest independent associations that may be over‐interpreted as causal or generalizable. Subgroup analyses are therefore presented as exploratory and hypothesis‐generating, rather than as estimates of independent predictors of care use.

For analysis, responses such as “don't know/not applicable” and “other” were treated as missing and excluded from percentage calculations, thus percentages refer to valid responses only. We considered a difference of ≥ 10% points (with *p* < 0.05) as clinically relevant, and we report only those subgroup differences meeting this threshold to focus on meaningful variations (given the large sample size, smaller differences could be statistically significant yet not practically important). No formal adjustment for multiple testing was applied, so the subgroup analyses should be interpreted with caution; however, our use of *a* ≥ 10% difference criterion was intended to reduce spurious emphasis on very small effects.

## 3 Results

### 3.1 Participants

**FIGURE 1:**
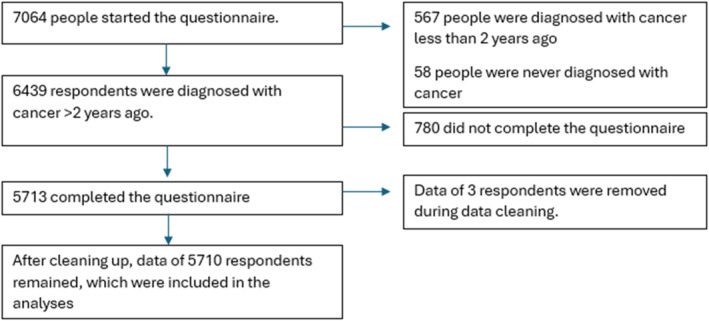
Flow chart.

This sample has been described previously, also see Figure 1 [14]. The majority were women (59%, *n* = 3346) and had a high education level (53%, *n* = 3016; Table 1). Two‐thirds of respondents (67%, *n* = 3831) were aged ≥ 60 years at the time of the questionnaire. The most common cancer types were breast (30%, *n* = 1706), hematological (25%, *n* = 1433), and prostate cancer (17%, *n* = 977). Furthermore, 61% (*n* = 3463) of participants were diagnosed before age 60%, and 68% (*n* = 3860) had been diagnosed more than 5 years ago. At the time of the survey, 26% (*n* = 1494) were still undergoing treatment, and nearly two‐thirds (64%, *n* = 3664) reported that they were (probably) cured or expected to be cured. Nearly half (44%, *n* = 2492) had received their care at a top clinical cancer hospital (as opposed to a general or university hospital).

**TABLE 1:** Sociodemographic and clinical characteristics of participants.

		*N*	%
Gender	Male	2353	41
	Female	3346	59
	Other/I prefer not to say	11	0
Education level^a^	Low	629	11
	Intermediated	1962	34
	High	3016	53
	I prefer not to say/different	103	2
Age at time of questionnaire	≤ 18 years	1	0
	19–39 years	182	3
	40–59 years	1696	30
	60–74 years	2758	48
	≥ 75 years	1073	19
Cancer type	Anal cancer	31	1
	Bladder or kidney cancer	166	3
	Blood or lymphatic cancer	1433	25
	Breast cancer	1706	30
	Colorectal cancer	206	4
	Gynecological cancer	265	5
	Brain tumor	65	1
	Head/neck cancer	111	2
	Lung cancer	194	3
	Gastric or esophageal cancer	169	3
	Melanoma or skin cancer	89	2
	Prostate cancer	977	17
	Sarcoma	62	1
	Thyroid cancer	35	1
	Testicular cancer	53	1
	Other	148	3
Age at diagnosis	≤ 18 years	41	1
	19–39 years	590	10
	40–59 years	2832	50
	60–74 years	2004	35
	≥ 75 years	243	4
Time since diagnosis	> 2–5 years ago	1850	32
	5–10 years ago	2289	40
	> 10 years ago	1571	28
Currently in treatment	Yes	1494	26
	Not anymore, but still follow‐ups	2786	49
	Not anymore, also no follow‐ups	1066	19
	No, never had, wait and see	139	2
	No, had treatment before, wait and see now	225	4
Disease phase	I (probably) don't have cancer anymore	3497	61
	I have cancer and will (probably) get better	167	3
	I have cancer and will (probably) not get better	884	16
	I have a chronic cancer type	912	16
	I have cancer and don't know if I will get better	250	4
Hospital	University medical center/cancer hospital	1754	31
	Top clinical hospital	2492	44
	General hospital	1261	22
	Other	203	2

^a^ Education level; (a) Low education: Primary or lower vocational education. (b) Intermediate education: Secondary general or vocational education. (c) High education: Higher professional or university education.

A significantly higher proportion of women (77%, *n* = 2586) were < 60 years at diagnosis compared to men (37%, *n* = 870). Additionally, women were more likely to report that they were probably (to be) cured (75%, *n* = 2462) than men (55%, *n* = 1192). Among those < 60 at diagnosis, 36% (*n* = 1242) had received their diagnosis > 10 years ago, compared to 15% (*n* = 329) of those aged ≥ 60. Finally, those diagnosed < 60 were more likely to state that they were or would be cured (73%, *n* = 2460) than those diagnosed ≥ 60 (58%, *n* = 1204).

Overall, the vast majority of respondents (approximately 89% (*n*∼5100)) reported experiencing at least one physical or mental health consequence of cancer or its treatment. The following results on care and support refer to those participants with self‐reported cancer‐related consequences unless stated otherwise.

### 3.2 Care or Support for the Consequences of Cancer

Among respondents with cancer‐related consequences, 33% (*n* = 1686) had received professional care or support for these issues in the past 3 months. Those receiving care were more likely to report certain symptoms than those not receiving care, such as fatigue 74% (*n* = 1240) versus 58% (*n* = 1967), decreased physical fitness 64% (*n* = 1068) versus 47% (*n* = 1595), lymphedema 25% (*n* = 428) versus 14% (*n* = 477), pain 32% (*n* = 536) versus 18% (*n* = 611), trouble moving 28% (*n* = 476) versus 16% (*n* = 534), sleep problems 34% (*n* = 567) versus 24% (*n* = 822), gloominess or depressive feelings 30% (*n* = 502) versus 17% (*n* = 583), trouble accepting (consequences of) cancer 31% (*n* = 514) versus 18% (*n* = 596), memory or concentration problems 45% (*n* = 753) versus 32% (*n* = 1071), problems with planning or keeping track 28% (*n* = 467) versus 17% (*n* = 570).

Respondents reporting the highest symptom burden (16–22 distinct symptoms) had the highest rate of professional care use (58%, *n* = 60), compared to 51% (*n* = 254) of those with 11–15 symptoms, 44% (*n* = 702) of those with 6–10 symptoms, and 24% (*n* = 649) of those with only 1–5 symptoms. Those 2–5 years post‐diagnosis were more likely to receive support (40%, *n* = 667) compared to those diagnosed 5–10 years (32%, *n* = 652) or over 10 years ago (27%, *n* = 367). Respondents (probably) not (getting) better were more likely to receive care (43%, *n* = 710) than those reported being cured or expecting to be cured (28%, *n* = 904). Respondents still in treatment at the time of the survey were more likely to be receiving care for consequences (50%, *n* = 715) than those not in treatment (27%, *n* = 971). No significant differences were found for gender, age at diagnosis, or education level.

### 3.3 Care or Support From Whom?

**FIGURE 2:**
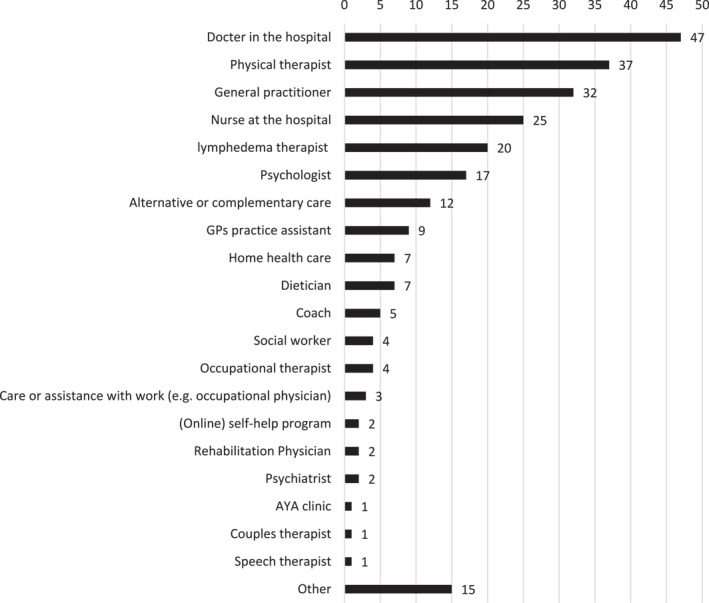
From whom do you receive professional care or support for the effects of (the treatment of) cancer.?

Among respondents receiving professional care for cancer consequences (*n* = 1686), the most commonly involved providers were medical specialists (47% of care‐recipients, *n* = 797), physical therapists (37%, *n* = 616), and general practitioners (32%, *n* = 538) (Figure 2).

Female care‐recipients were more likely to use certain services than male care‐recipients. For example, 43% of women (*n* = 447) receiving care saw a physical therapist, compared to 27% of men (*n* = 169). Similarly, women more often reported seeing a lymphedema therapist (28% vs. 5% of men), a psychologist (21% vs. 11%), or an alternative/complementary care provider (16% vs. 6%). Conversely, men who were receiving care more often did so with a medical specialist (63% vs. 38% of women).

Respondents < 60 years at diagnosis were more likely to receive care or support from a physical therapist (40%, *n* = 434 vs. 30%, *n* = 182), lymphedema therapist (26%, *n* = 285 vs. 8%, *n* = 46), psychologist (22%, *n* = 238 vs. 9%, *n* = 55) and alternative or complementary care (16%, *n* = 169 vs. 6%, *n* = 36) than those ≥ 60. The older group was more likely to receive care from the general practitioner (38%, *n* = 229 vs. 28%, *n* = 309), medical specialist (61%, *n* = 365 vs. 40%, *n* = 432) and hospital nurse (32%, *n* = 189 vs. 21%, *n* = 233).

Respondents 2–5 years post‐diagnosis reported hospital nurse support more often (30%, *n* = 201) than those 5–10 years (24%, *n* = 155) or > 10 years post‐diagnosis (18%, *n* = 66).

Among those receiving care, respondents (probably) not (getting) better were more likely to report receiving care or support from their general practitioner (39%, *n* = 273 vs. 26%, *n* = 239), medical specialist (64%, *n* = 456 vs. 33%, *n* = 294) and hospital nurse (35%, *n* = 247 vs. 17%, *n* = 157) than those reported being cured or expecting to be cured. Conversely, respondents who considered themselves cured or likely to be cured were more likely to use a lymphedema therapist (32%, *n* = 287) than those in the not‐(getting)‐better group (6%, *n* = 39).

Respondents not in treatment were more likely (25%, *n* = 245) to have received care or support from the lymphedema therapist than respondents currently in treatment (12%, *n* = 86). Those currently in treatment reported receiving care or support from their medical specialist (59%, *n* = 421 vs. 39%, *n* = 376) and nurse in the hospital (36%, *n* = 259 vs. 17%, *n* = 163) more often.

Highly educated respondents (15%, *n* = 132) were more likely to use alternative or complementary care or support than middle (10%, *n* = 60) and low‐educated (4%, *n* = 7) participants.

### 3.4 No Care or Support, But Would Like it

Among respondents with cancer‐related consequences, 15% (*n* = 735) indicated that they were not receiving professional care for those issues despite wanting it. An additional 21% (*n* = 1052) were unsure whether they needed care, and 64% said they did not need care for their cancer/treatment‐related problems. No significant differences were found across gender, age at diagnosis, time since diagnosis, disease stage, treatment stage, or education level.

**FIGURE 3:**
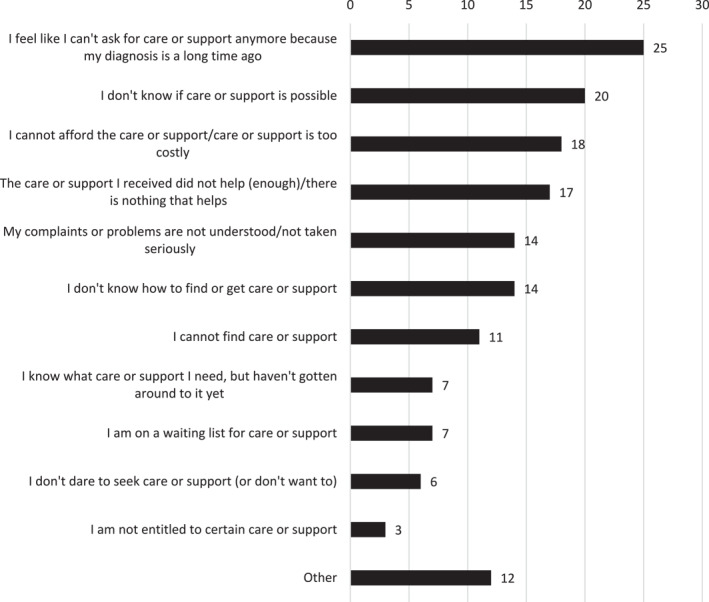
Why didn't you receive professional care or support for the consequences (from the treatment of) cancer.?

Among respondents with an unmet desire for care (*n* = 735), the most common reasons were: feeling unable to ask for support because so much time had passed since diagnosis (25%, *n* = 177), not being sure if any care was available for their issues (20%, *n* = 143), and not being able to afford care (18%, *n* = 130) (Figure 3). Women in this unmet‐need group were more likely than men to cite cost as a barrier (23% of female respondents vs. 12% of males reporting they “couldn't afford it”).

Of those who felt they could no longer ask for care due to the length of time since diagnosis, 30% (*n* = 147) of respondents diagnosed before 60 felt this way, compared to 19% (*n* = 30) of those diagnosed at 60 or older. Respondents whose diagnosis was > 10 (33%, *n* = 47) or between 5 and 10 years (35%, *n* = 98) ago were more likely to feel they couldn't ask for care compared to those diagnosed 2–5 years ago (14%, *n* = 32). Respondents who reported being cured or expecting to be cured (31%, *n* = 137) and those not in treatment (31%, *n* = 143) were more likely to feel they could no longer ask for care than those (probably) not (getting) better (18%, *n* = 34) or currently in treatment (18%, *n* = 34).

Notably, among respondents with unmet needs, those with low education were more likely to report not knowing how to find appropriate care or support (30%, *n* = 13) compared to those with intermediate (18%, *n* = 37) or high education (12%, *n* = 44).

### 3.5 Clear Who to Turn to?

Overall, about two‐thirds of respondents (68%, *n* = 3886) reported that they knew which professional to turn to for help with new or ongoing consequences of cancer, whereas 19% (*n* = 1061) did not know and 13% (*n* = 763) were unsure. Highly educated survivors were more likely to have clarity (74%, *n* = 2226) than those with intermediate (64%, *n* = 1250) or low education (55%, *n* = 346) (*p* < 0.05), with no significant differences by gender, age at diagnosis, time since diagnosis, disease phase, or treatment phase.

### 3.6 Support or Help From Peers or Volunteers

Among respondents with cancer‐related consequences, 19% (*n* = 967) received support from peers or volunteers in the last 3 months, while 10% (*n* = 505) wanted but did not receive such support. The remaining 71% (*n* = 3610) did not receive nor need this type of help.

Respondents diagnosed 2–5 years ago were more likely (24%, *n* = 400) to receive support from peers or volunteers than those diagnosed 5–10 years (19%, *n* = 383) or over 10 years ago (14%, *n* = 184). No significant differences were found based on gender, age at diagnosis, disease stage, treatment stage, or education level.

## 4 Discussion

About one‐third of surveyed survivors experiencing cancer‐related consequences more than 2 years post‐diagnosis, were receiving professional care or support at the time of the survey, and even among those > 10 years from diagnosis, over one‐quarter were still receiving such care. Conversely, a substantial subset of survivors with ongoing consequences reported wanting support but not receiving it (∼15%, as noted). These findings underscore the ongoing need for accessible care and support for long‐term cancer survivors, as persistent (late) sequelae can continue to impact quality of life [15].

We found that professional care for cancer's consequences was most often delivered by medical specialists, physical therapists, and general practitioners, reflecting the multidisciplinary nature of survivorship care. Survivors with a higher symptom burden, those 2–5 years post‐diagnosis, those (probably) not (getting) better, and those still in active treatment were the most likely to be receiving support, indicating that survivors with ongoing or more severe issues engage with care more frequently. This pattern underlines that many treatment‐related symptoms can persist or resolve only gradually, so care for cancer's after‐effects is often needed even years after the initial diagnosis [4].

Since many long‐term treatment consequences cannot be fully prevented, it is important that healthcare providers proactively discuss potential late effects with patients and engage in shared decision‐making before treatment begins [16]. For example, one in five respondents who desired care did not know if it was available for them, and others felt they could no longer ask due to the time elapsed since diagnosis. These patterns indicate a lack of ongoing survivorship follow‐up and insufficient communication about long‐term consequences and available care pathways. This confirms the literature that states that poor care coordination remains a significant challenge, with survivors reporting fragmentation in care and uncertainty about post‐treatment management [17, 18]. Limited patient education is identified as a key barrier, with studies emphasizing the importance of knowledge and education in facilitating successful post‐treatment care [19]. Unclear referral processes and inadequate care coordination are persistent issues [17]. While survivorship care plans (SCPs) are widely recommended, their effectiveness in addressing these barriers is limited. Studies show no significant impact of SCPs on cancer‐care coordination [20]. However, SCPs may improve survivors' ability to identify responsible clinicians and potentially reduce unmet needs [20]. Moreover, affordability concerns and lower health literacy, especially among lower educated respondents, also contribute to unmet needs. This suggests that the unmet needs observed are due to a combination of factors: limited awareness, unclear referral structures, financial barriers, and inconsistent integration of survivorship care into long‐term follow‐up. Considering limited healthcare resources, prioritizing interventions that address these systemic and informational gaps, rather than expanding care indiscriminately, is essential. Ensuring that patients are informed about what to expect may help them cope better with any long‐term consequences that do arise. Furthermore, systematically monitoring PROs throughout the cancer care continuum can facilitate timely identification and management of persistent symptoms.

In the Netherlands, a broad range of supportive care services is covered by the mandatory basic health insurance (e.g., GP consultations, specialist oncology or rehabilitation care, hospital nursing, and physician‐referred psychological services). Physical therapy is only reimbursed in full after the 20th session for chronic conditions, though supplementary insurance can bridge some gaps. Non‐medical support (e.g., social work, home assistance) is funded at the municipal level through the Social Support Act (WMO). Despite this extensive coverage, out‐of‐pocket costs (due to deductibles, co‐pays, or uncovered services) can still pose financial barriers for survivors seeking care.

In the context of the Dutch healthcare system, our findings highlight several areas for improvement to better support long‐term survivors. We do not suggest a radical overhaul of the system since the current infrastructure generally provides broad access to care, especially for those with adequate insurance coverage [11]. However, targeted enhancements are needed to close persistent gaps. For instance, some survivors in our survey lacked awareness of available support options or hesitated to seek help because many years had passed since diagnosis. Lower‐educated respondents were particularly more likely to not know where to turn with new symptoms, reflecting a health literacy gap. This echoes findings from other settings that limited health literacy is a significant barrier to accessing care [21]. Improving communication about survivorship care is therefore critical.

Health care providers, community services, and patient organizations should collaborate to clearly and proactively inform survivors about where they can go for which type of issue, even long after treatment. Patient organizations (like those under NFK) can play a guiding role. For example, the online platform Kanker.nl allows patients to search for nearby support by symptom or provider, and it provides information on municipal support services for those who cannot afford certain care. Nevertheless, not all patients are adept with online resources, and some may still miss this information. Ongoing efforts by NFK and its member organizations will focus on making information and care pathways more accessible, including strengthening the role of informal care and social support networks, to ensure survivors of all backgrounds can obtain the help they need. These recommendations are tailored to the Dutch context, where leveraging existing robust insurance and community support structures (rather than overhauling them) could address the identified informational and coordination gaps.

Our findings also underscore the importance of encouraging survivors to promptly address new or persistent symptoms, no matter how long it has been since diagnosis, by seeking advice from healthcare providers. Even years after completing regular follow‐up at the hospital, survivors can consult their general practitioner or contact hospital‐based care coordinators to discuss any late‐emerging issues [22]. There may be more support options available than many survivors realize, and early communication can help identify interventions or management strategies that improve quality of life. Patient organizations and resources like Kanker.nl offer extensive information on late effects of cancer and available support services (including peer and volunteer support), which might alleviate symptoms or can empower survivors to seek professional help when needed.

In summary, several actions could be considered. First, we advocate for increased awareness and clarity, not only among patients but also among healthcare professionals, about the availability and relevance of professional and peer support services for long‐term cancer consequences. Awareness could be raised about available services and resources, particularly for those who may not be aware of, or have difficulty navigating, the healthcare system. Second, to improve accessibility to affordable professional care, mechanisms such as improved insurance coverage should be explored. Furthermore, one could expand online services to reach those with mobility challenges or to lower the bar for obtaining help. Next, more attention could be raised to peer support networks, both in‐person and online. Additionally, volunteer programs that allow trained individuals to provide emotional and practical support to patients could be promoted. Moreover, personalized follow‐up care could ensure that emotional, psychological, and social needs are addressed alongside physical health, also long after diagnosis. Finally, health systems should integrate survivorship care planning into routine oncology care, with clear guidelines on who is responsible for long‐term symptom management [23]. Also, information provision to both patients and healthcare professionals on seeking support for cancer consequences, whether it's professional care or peer connections could be helpful. By focusing on these areas, we can help ensure that more cancer patients receive the support they need, both professionally and from their peers.

This study benefits from a high response rate, providing valuable insights into the experiences of individuals living with or beyond cancer. Several limitations of our study should be noted. Our sample's demographics differ somewhat from the broader survivor population. For example, the women in our study were, on average, younger than the men, which may confound some sex‐specific comparisons. Education level was notably higher in our respondent group than in the general population of Dutch cancer survivors; 53% of our participants had high education versus about 36% in national population data (2023). Low‐educated survivors were underrepresented (11% in our sample vs. ∼26% nationally). This overrepresentation of highly educated, potentially more health‐literate individuals could bias our findings: it might inflate reports of proactive health behaviors or awareness (and conversely understate the challenges faced by lower‐educated survivors). We did have a substantial number of lower‐educated respondents (*n* = 629), enabling subgroup analysis, but the skewed education profile still limits generalizability of certain results.

Our sample's cancer‐type mix also differed from national patterns: breast and prostate cancer survivors were overrepresented (consistent with their overall prevalence), and hematological cancer survivors were also relatively numerous, whereas survivors of lung, gastrointestinal, and gynecological cancers were fewer than expected. This imbalance could skew certain results. For instance, symptoms common in overrepresented groups (e.g., prostate cancer‐related issues like urinary or sexual symptoms) might appear more prevalent in our overall results, while issues typical of underrepresented groups could be underestimated. Readers should thus be cautious in generalizing symptom frequency findings to all cancer survivors, as they may reflect our sample's composition.

Furthermore, our survey instrument, while developed with extensive patient and expert input to ensure content validity, did not undergo a formal pilot test. This means we did not quantitatively pre‐validate the questionnaire (e.g., through psychometric analysis or cognitive interviews), which could have identified issues in question interpretation. However, the iterative design process with a diverse team was intended to mitigate this limitation. We also lacked data on participants' comorbid conditions; such information might have been relevant, as other health issues could influence a survivor's need for or use of care. Also, health care use was assessed using a 3‐month recall period. While this approach reduces recall bias and reflects current care use, it does not capture cumulative, intermittent, or episodic care over longer survivorship trajectories. Consequently, longer‐term health care use may be underestimated, and our findings should be interpreted as a snapshot of recent care engagement rather than a comprehensive overview of survivorship care patterns. In addition, although the survey included open‐ended questions, we did not conduct a formal thematic analysis of these responses. Incorporating qualitative analysis with exemplar quotations could provide richer insight into survivors' experiences, motivations, and perceived barriers to care, and may help contextualize the quantitative patterns observed.

Another key limitation is selection bias. Participation was voluntary and the survey was broadly promoted, which likely means that survivors currently experiencing issues (or those particularly engaged with survivorship topics) were more inclined to respond. Those who have moved on from cancer and have no ongoing problems may have been underrepresented. Additionally, the online‐only format inherently excludes or discourages some groups, notably survivors with limited internet access or skills, often older individuals. This is significant because older survivors (who might be under‐sampled here) have distinct needs that could be under‐reflected in our results [24]. The strong involvement of patient organizations in study design and recruitment also influences the sample. Because the survey was distributed largely via patient advocacy channels, a substantial portion of respondents were likely members of patient organizations or followers of related social media. These participants might be more proactive, health‐conscious, or informed about their condition than the average survivor, introducing a perspective bias in favor of the engaged patient. We attempted to mitigate this by wording the invitation broadly (e.g., explicitly encouraging participation even from those for whom cancer is not a current issue), and by distributing the survey through some hospitals and the Dutch Cancer Society to reach beyond the advocacy community. Nonetheless, our sample is probably skewed toward more engaged and empowered survivors, so general caution is warranted in extrapolating the absolute levels of awareness or unmet needs to all survivors. Finally, given the cross‐sectional design of our survey, all data were captured at a single time point. We cannot establish causal relationships or determine the directionality of associations observed (e.g., whether receiving professional support leads to better outcomes, or whether those with worse symptoms are simply more likely to seek support). Nor can we track changes in individual survivors' needs over time with these data. Our results should thus be interpreted as associative snapshots rather than evidence of causation.

In summary, our survey reveals that even after treatment, many survivors continue to grapple with physical, psychological, and cognitive challenges, and a notable fraction do not receive the help they desire. These findings underscore the need for a coordinated, multidisciplinary survivorship care approach that addresses not only medical issues but also rehabilitation, psychosocial support, and patient education. By identifying specific gaps in support (especially for certain subgroups), our study provides direction for improving survivorship care within the Dutch healthcare system and perhaps in similar health systems elsewhere.

## Author Contributions

**Floortje Mols:** conceptualization, writing – original draft, writing – review and editing. **Noortje van Willegen:** conceptualization, writing – review and editing. **Dagna Lek:** writing – review and editing. **Vivian Engelen:** conceptualization, data collection, writing – review and editing.

## Funding

The authors have nothing to report.

## Conflicts of Interest

The authors declare no conflicts of interest.

## Data Availability

Data were collected for the purpose of this study. Data are securely stored and can be viewed at the Dutch Federation of Cancer Patient Organizations (NFK). Restrictions apply to the use of this data.
